# Prostate Cancer Screening: Current Controversies and Future Directions

**DOI:** 10.7759/cureus.110934

**Published:** 2026-06-15

**Authors:** Hasan F Buali, Abdulaziz Al Shaibani, Umar S Farouqi, Tarek Abushloa, Ahmed Bastawisy, Noora Aljeeran, Khalifa Albenjasim, Mohamed Rafie

**Affiliations:** 1 Urology, King Hamad University Hospital, Muharraq, BHR; 2 Urology, Bahrain Defence Force Hospital, Riffa, BHR; 3 Urology, king hamad university hospital, Muharraq, BHR

**Keywords:** early detection, overdiagnosis, prostate cancer, prostate cancer screening, prostate-specific antigen (psa)

## Abstract

Prostate-specific antigen (PSA) testing has been central to prostate cancer detection since FDA approval for early detection in 1994, yet its widespread application has generated enduring controversy owing to limited specificity, overdiagnosis, and the quality-of-life harms of overtreatment. Prostate cancer is among the most prevalent malignancies in men globally. Major randomized trials, including the European Randomized Study of Screening for Prostate Cancer (ERSPC), the Prostate, Lung, Colorectal, and Ovarian (PLCO) Cancer Screening Trial, and the more recent ProtecT trial, reported divergent conclusions about prostate cancer-specific mortality benefit. International guidelines from the American Urological Association/Society of Urologic Oncology (AUA/SUO), the United States Preventive Services Task Force (USPSTF), and the European Association of Urology (EAU) differ substantially in their recommended age thresholds, screening intervals, and approaches to shared decision-making, reflecting the genuine uncertainty in the evidence base.

Emerging modalities, such as multiparametric magnetic resonance imaging, serum biomarkers including the Prostate Health Index and 4Kscore, and germline genomic risk stratification, offer pathways toward more individualized screening. Polygenic risk scores and artificial intelligence-based tools show promise for integrating multiple risk factors, though most approaches remain under active investigation and require prospective validation before routine clinical deployment. This narrative review synthesizes current evidence on PSA-based screening, evaluates the principal sources of controversy, compares international guideline recommendations, and examines emerging risk-stratified approaches. Current evidence supports individualized, risk-adapted screening over universal PSA threshold application, with shared decision-making as the essential framework for integrating emerging biomarker and genomic data into clinical practice.

## Introduction and background

Prostate cancer is a major global public health burden, ranking as the second most frequently diagnosed malignancy in men worldwide and the fifth leading cause of cancer-related death in men globally [1]. In 2022, an estimated 1,467,854 new cases were recorded, with incidence disproportionately elevated in Western nations, among men of African ancestry, and in older age groups [1,2]. The disease spans a wide biological spectrum, from indolent, low-grade tumors unlikely to cause symptoms during a patient's lifetime, to aggressive, metastatic phenotypes associated with poor prognosis [3].

Prostate-specific antigen (PSA) testing was introduced as a blood-based screening marker in the late 1980s and early 1990s, fundamentally altering the epidemiology of prostate cancer detection [4,5]. The FDA approved PSA for monitoring in 1986 and for early detection in 1994; its clinical adoption was subsequently rapid and widespread, attributable to its non-invasive nature as a simple blood test, low cost, and high sensitivity for early-stage disease [4,6]. However, the years that followed revealed a paradox: while incidence rates surged and mortality rates declined, modeling studies reported substantial uncertainty about how much of this reduction was attributable to screening versus improvements in treatment, and how much increased incidence represented overdiagnosis [7].

Major randomized trials including the European Randomized Study of Screening for Prostate Cancer (ERSPC) trial and the Prostate, Lung, Colorectal, and Ovarian (PLCO) trial produced conflicting results [8,9], and international guidelines remain discordant [10-12]. Critically, much of this evidence derives from organized population-based screening programs, which differ fundamentally from the individual opportunistic testing that occurs when a patient presents to a physician seeking evaluation - a distinction with direct implications for clinical practice. Emerging advances in imaging, biomarkers, genomics, and artificial intelligence (AI) may refine risk stratification and improve the precision of early detection, although many approaches still require prospective validation before routine deployment [13,14].

This narrative review examines the historical trajectory of PSA screening, synthesizes evidence on current and emerging screening modalities, analyzes the principal sources of controversy, and evaluates future directions in prostate cancer early detection.

Methods

A systematic search of PubMed was conducted using the following terms: "prostate cancer screening", "prostate-specific antigen", "PSA screening", "multiparametric MRI prostate", "prostate cancer biomarkers", "polygenic risk score prostate cancer", and "artificial intelligence prostate cancer diagnosis". Searches were limited to English-language articles published up to March 2025. Additional relevant studies were identified through a manual review of reference lists of the retrieved articles. Priority was given to randomized controlled trials, systematic reviews, meta-analyses, and current clinical practice guidelines. Studies were included if they addressed PSA-based screening outcomes, guideline recommendations, emerging detection modalities, or risk stratification strategies. Non-English publications, conference abstracts, and studies without peer-reviewed full-text availability were excluded.

## Review

Historical perspective on PSA screening

The molecular foundation of PSA-based screening was established in 1979, when Wang et al. first purified PSA from prostatic tissue [15]. Stamey et al. subsequently reported in 1987 that PSA could serve as a serum marker for adenocarcinoma of the prostate [6]. The pivotal shift toward diagnostic screening came in 1991, when Catalona et al. reported that PSA measurement improved prostate cancer detection compared with digital rectal examination (DRE) alone [4]. These findings preceded FDA approval for early detection in 1994 [4].

Following FDA approval, PSA testing was rapidly incorporated into routine health maintenance for men aged over 50 years. The epidemiological impact was immediate: prostate cancer incidence approximately doubled between the mid-1980s and early 1990s, while the proportion of men diagnosed with distant-stage disease declined markedly, consistent with a stage-migration effect attributable to earlier detection [4,6]. However, autopsy series consistently reported that a substantial proportion of older men harbor histological prostate cancer that never causes symptoms or contributes to death, establishing the biological basis for overdiagnosis risk associated with population-wide screening [16].

Two landmark randomized controlled trials were initiated to determine whether PSA screening reduces prostate cancer-specific mortality. The ERSPC, enrolling over 162,000 European men aged 55 to 69, reported a 21% reduction in prostate cancer-specific mortality after 13 years of follow-up, with an estimated 781 invitations and 27 additional prostate cancer diagnoses required to prevent one prostate cancer death [8]. In contrast, the PLCO trial, which enrolled over 76,000 American men, found no significant reduction in prostate cancer mortality with organized screening at 13 years of follow-up [9]. The divergence between these trials has been partly explained by modeling studies suggesting that high rates of PSA testing in the PLCO control arm - estimated contamination exceeding 80% - substantially attenuated any detectable mortality difference [7].

Current screening modalities

Digital Rectal Examination

DRE represents the most fundamental component of the clinical prostate examination and historically preceded PSA testing as the primary screening modality. DRE provides information about prostatic consistency and contour, and its combination with PSA improves the detection of clinically significant disease compared with PSA alone [4]. Contemporary guidelines position DRE primarily as an adjunct to PSA measurement rather than an independent screening test [11,12].

Prostate-Specific Antigen Testing

PSA remains the cornerstone of prostate cancer screening. PSA, a glycoprotein produced almost exclusively by prostate epithelial cells, increases in states of prostate enlargement, inflammation, and malignancy [3]. Conventionally, a serum PSA threshold of 4 ng/mL has been used to trigger further evaluation, although the European Association of Urology (EAU) recommends consideration of biopsy at approximately 3 ng/mL in eligible individuals [11]. PSA density, calculated as serum PSA divided by prostate volume as measured by ultrasound, can improve risk assessment by contextualizing PSA in relation to gland size [17]. PSA velocity, the rate of PSA change over time, has been proposed as an additional adjunct; however, its independent utility beyond absolute PSA and PSA density remains debated, with a systematic review finding insufficient evidence to support its use as a standalone metric [18].

Despite its widespread use, PSA's principal limitations are well-established. Specificity is limited, and many biopsies prompted by elevated PSA historically have not detected cancer, reflecting PSA's sensitivity to benign conditions including benign prostatic hyperplasia and prostatitis [18]. PSA circulates in two forms: free (unbound) and complexed (bound to proteins such as alpha-1-antichymotrypsin). The ratio of free-to-total PSA improves specificity because benign prostatic conditions tend to elevate free PSA proportionally more than malignancy does; consequently, a lower free-to-total ratio indicates a higher probability of prostate cancer and is widely used as a reflex test after initial PSA elevation [3].

Multiparametric Magnetic Resonance Imaging

Multiparametric magnetic resonance imaging (mpMRI) of the prostate has emerged as a transformative tool in the pre-biopsy pathway. It combines T2-weighted, diffusion-weighted, and dynamic contrast-enhanced sequences, with reporting standardized by the Prostate Imaging Reporting and Data System (PI-RADS v2.1). The PROMIS trial reported that mpMRI could allow approximately 27% of men to avoid immediate biopsy, achieving 93% sensitivity for clinically significant cancer (defined as Gleason score ≥4+3 or maximum cancer core length ≥6 mm) [19]. The PRECISION trial subsequently showed that MRI-targeted biopsy detected more clinically significant cancers and fewer clinically insignificant cancers compared with standard systematic biopsy [20]. The EAU now recommends pre-biopsy mpMRI in men with suspected prostate cancer before a first biopsy is performed [11]. Cost, access, and inter-reader variability remain important barriers.

Blood-Based Biomarkers: PHI, 4Kscore, and Others

Several blood-based biomarkers have been developed to stratify biopsy risk more accurately than PSA alone, particularly in the PSA range of 2-10 ng/mL. The Prostate Health Index (PHI) combines total PSA, free PSA, and the [−2]proPSA isoform; prospective studies have reported that PHI can reduce unnecessary biopsies by approximately 30-41% while maintaining sensitivity for clinically significant disease [21].

The 4Kscore combines four kallikrein markers - total PSA, free PSA, intact PSA, and human kallikrein-related peptidase 2 (hK2) - with clinical variables including age, DRE result, and prior biopsy status. In a prospective multi-institutional trial, the 4Kscore reported an area under the curve of 0.82 for high-grade cancer detection; applying a 7.5% risk threshold reduced unnecessary biopsies by approximately 30% without missing high-grade cancers [22]. Combined approaches using mpMRI with kallikrein-based biomarkers may further improve discrimination [23]. Urine-based biomarkers including PCA3 (prostate cancer antigen 3) add incremental information beyond PSA primarily in the context of repeat biopsy decisions [24].

It should be emphasized that none of these biomarkers - PHI, 4Kscore, or PCA3 - are currently recommended by the American Urological Association/Society of Urologic Oncology (AUA/SUO), EAU, or United States Preventive Services Task Force (USPSTF) as independent population-level screening tools; their role is confined to risk stratification and biopsy decision-making in men with already elevated PSA [10-12].

Economic Considerations

The economic implications of prostate cancer screening warrant consideration. PSA testing itself is low-cost, but the downstream consequences of screening, including diagnostic workup, biopsy, active surveillance (AS) protocols, and definitive treatment, generate substantial healthcare expenditure. Emerging tools such as mpMRI, PHI, and the 4Kscore carry higher per-test costs than PSA alone, though health economic analyses suggest that their selective use to reduce unnecessary biopsies may offset downstream costs in certain healthcare systems. Polygenic risk score (PRS) testing and AI-assisted diagnostics remain largely investigational, and their cost-effectiveness has not been established across diverse healthcare settings. Equitable access to advanced screening modalities represents an important unresolved challenge, particularly in lower-resource environments where even PSA testing may not be universally available [11,12].

Controversies and debates

Overdiagnosis and Overtreatment

Overdiagnosis, defined as the identification of cancers that would never have become clinically evident during a patient’s lifetime, represents the most fundamental challenge of prostate cancer screening. Estimates of overdiagnosis attributable to PSA screening vary widely, reflecting differences in population characteristics, PSA thresholds, biopsy criteria, and modeling assumptions [16]. Lead-time estimates have been reported to range from several to over 10 years in different cohorts [16].

The consequences of overdiagnosis are compounded by overtreatment. Radical prostatectomy is associated with the highest rates of urinary and sexual dysfunction, while radiotherapy carries greater rates of bowel dysfunction; importantly, radiotherapy-associated complications, including bowel, urinary, and sexual sequelae, may manifest or progress decades after treatment, a consideration of increasing clinical relevance as life expectancy continues to rise globally. Both treatment modalities affect quality of life in ways that may be disproportionate to the oncological benefit for men with indolent disease [25]. The ProtecT trial reported that at 10 years, prostate cancer-specific mortality was similarly low across active monitoring, surgery, and radiotherapy arms in men with PSA-detected localized disease [26]. At 15 years, prostate cancer-specific mortality remained low across all groups - 2.7% in the active monitoring group, 3.1% in surgery, and 3.3% in radiotherapy, differences that were not statistically significant - although men assigned to active monitoring experienced higher rates of metastatic disease (9.4% vs 4.7% for surgery and 5% for radiotherapy) [27].

AS has emerged as the primary strategy for managing low-risk screen-detected cancers without immediate treatment. AS is now recommended by all major guidelines for Grade Group 1 (Gleason 6) and selected low-volume Grade Group 2 cancers [11,12]. The concept of competing mortality risk is central to these decisions: for older men or those with significant comorbidities, the probability of dying from a cause other than prostate cancer within ten years may substantially exceed the risk of prostate cancer-specific death, further reinforcing the importance of individualized rather than age-based screening and treatment thresholds [11,12]. Patient anxiety, adherence to monitoring protocols, and variability in AS definitions across institutions remain implementation challenges.

Population-Based Screening Versus Individual Opportunistic Testing

A fundamental distinction that is frequently conflated in the prostate cancer screening debate is the difference between organized population-based screening programs and individual opportunistic testing in clinical practice. Population-based screening involves the systematic invitation of all men within a defined age range, regardless of symptoms or risk factors, with the aim of reducing disease-specific mortality at a population level. The major randomized trials ERSPC and PLCO were designed to evaluate this model, and their findings, including modest mortality benefit, high numbers needed to screen, and significant overdiagnosis rates, apply specifically to this context [7-9].

Individual opportunistic testing, by contrast, occurs when a patient presents to a physician for evaluation or routine care and, following an informed discussion, elects to undergo PSA testing. This scenario differs fundamentally from population screening in several clinically important ways. The patient has already self-selected by seeking medical attention, which itself may reflect symptom awareness, family history, health-seeking behavior, or elevated personal risk perception. The clinical encounter provides the opportunity for genuine shared decision-making (SDM), individualized risk assessment, and contextual interpretation of results in a way that population invitation programs cannot replicate [10-12].

The major international guidelines recognize this distinction, albeit implicitly. The USPSTF Grade C recommendation applies to population-level screening decisions and specifically endorses individualized decision-making for men aged 55 to 69 [10]. The AUA/SUO guidelines similarly frame their recommendations around the clinical encounter between an informed patient and physician rather than population-wide invitation programs [12]. The EAU adopts an early detection rather than screening paradigm, positioning PSA testing as a risk-adapted tool for men who present seeking evaluation rather than a universal population intervention [11].

In clinical practice, the evidence base from population trials should inform but not dictate individual patient decisions. A man in his mid-fifties with a family history of prostate cancer who presents to his urologist requesting PSA testing occupies a fundamentally different position than an anonymous member of a population screening cohort. For such individuals, the benefit-harm calculation shifts considerably: baseline risk is higher, the clinical context enables more nuanced interpretation, and the patient's own values and preferences - elicited through SDM - appropriately guide whether and how testing proceeds. Failing to distinguish these two contexts risks applying population-level conclusions inappropriately to individual patients, potentially denying testing to men who would meaningfully benefit from early detection.

Divergent International Guidelines

The most visible expression of this controversy is the discordance among major guideline-issuing bodies. In 2012, the USPSTF issued a Grade D recommendation against PSA-based screening for all men [28]. This recommendation coincided with a measurable decline in screening rates and has been associated in retrospective analyses with subsequent increases in the proportion of men presenting with advanced-stage disease [10]. In 2018, the USPSTF revised its guidance to a Grade C recommendation for men aged 55 to 69 years, acknowledging a small net benefit when decisions are individualized through SDM, while maintaining a recommendation against screening in men aged 70 years or older [10].

The AUA/SUO 2023 guideline takes a more affirmative stance, classifying screening every two to four years for men aged 50 to 69 years as a Strong Recommendation with Grade A evidence, and recommending earlier discussion from age 45 for average-risk men, and at 40 to 45 years for those at higher risk [12]. The EAU 2024 guidelines adopt a risk-adapted early detection strategy, recommending PSA-based screening in well-informed men at increased risk, with a biopsy threshold of approximately 3 ng/mL and testing discouraged in men with a life expectancy below 15 years [11]. Notably, the 2024 update reinforces pre-biopsy mpMRI as standard of care and incorporates germline testing recommendations for high-risk individuals, reflecting advances since the previous iteration. Table 1 provides a structured comparison of these guideline recommendations.

**Table 1:** Comparison of major international prostate cancer screening guideline recommendations AUA/SUO, American Urological Association/Society of Urologic Oncology; EAU, European Association of Urology; DRE, digital rectal examination; mpMRI, multiparametric magnetic resonance imaging; PSA, prostate-specific antigen; USPSTF, United States Preventive Services Task Force

Parameter	AUA/SUO 2023 [12]	USPSTF 2018 [10]	EAU 2024 [11]
Screening test	PSA (primary); secondary biomarkers as adjuncts	PSA-based	PSA ± DRE; biopsy threshold ~3 ng/mL
Age to start (average risk)	45–50 years	55–69 years (Grade C)	≥50 years (well-informed)
Age to start (high risk)	40–45 years (African ancestry, BRCA2, family history)	Not specified separately	≥45 (family history/African descent); ≥40 (BRCA2)
Screening interval	Every 2–4 years (ages 50–69)	Periodic; interval not specified	Risk-adapted (individualized)
Age to stop	Individualized; life expectancy and preference	≥70 years: recommend against	<15-year life expectancy: discouraged
Shared decision-making	Emphasized for all ages	Core requirement for 55–69	Mandatory counselling before testing
Recommendation grade	Strong Recommendation; Grade A (ages 50–69)	Grade C (small net benefit in selected men)	Moderate recommendation
Role of MRI	Recommended prior to biopsy	Not prominently addressed	Pre-biopsy mpMRI recommended

Shared Decision-Making: Aspirational Standard or Practical Challenge?

All contemporary guidelines converge on SDM as the appropriate framework for PSA screening decisions. SDM requires clinicians to discuss potential benefits of early detection, the likelihood that a detected cancer would threaten health if undetected, the risk of false-positive results and associated biopsy complications, and the potential adverse effects of treatment [10-12]. In practice, SDM faces significant implementation barriers in primary care: time constraints, variable clinician familiarity with evolving evidence, and health literacy disparities may compromise the quality of SDM conversations [29].

Racial and Ethnic Disparities

Men of African ancestry experience substantially higher rates of prostate cancer incidence and mortality than white men in the United States [1,2]. These disparities likely reflect structural factors including unequal access to screening and treatment, socioeconomic barriers, differential representation in clinical trials, and possible tumor-biologic differences [2]. Contemporary guidelines from the AUA/SUO and EAU explicitly classify African ancestry as a high-risk factor warranting discussion of earlier screening initiation [11,12]. Ensuring that advanced risk-stratification tools including PRSs and AI models are validated in diverse populations is essential to avoid amplifying existing inequities [30].

Emerging approaches

Risk-Stratified and Precision Screening

Risk-stratified screening aims to direct intensive early detection efforts toward men with the highest probability of harboring clinically significant cancer. The Stockholm3 (STHLM3) model integrates protein biomarkers and common genetic variants alongside clinical variables. In a prospective population-based trial, STHLM3 reported a 32% reduction in the number of biopsies performed compared with PSA alone, while maintaining equivalent detection of clinically significant prostate cancer (Gleason Grade Group 2 or higher) [31]. These findings have been replicated in additional validation cohorts, though performance varies by population tested and definition of clinical significance applied [31].

Germline Genetics and Polygenic Risk Scores

Prostate cancer has one of the highest heritability estimates among common cancers, with approximately 57-58% of disease risk attributable to heritable factors in large Nordic twin studies [32]. Rare pathogenic variants in genes including BRCA2, ATM, CHEK2, mismatch repair genes (MSH2/MSH6), and HOXB13 are associated with increased prostate cancer risk. Germline testing is recommended by major guidelines in selected high-risk settings, including men with metastatic prostate cancer and those with suggestive family histories [11,12,33].

Genome-wide association studies have identified several hundred common genetic variants associated with prostate cancer susceptibility, which can be aggregated into PRSs reflecting an individual's cumulative genetic predisposition [33]. The BARCODE1 study reported that offering intensive PSA-based screening selectively to men in the highest decile of polygenic risk identified prostate cancer in approximately 40% of biopsied participants, with more than half meeting intermediate- or high-risk criteria by NCCN 2023 standards [34]. While PRS-guided approaches offer a conceptually compelling route to improved cancer yield per biopsy, the vast majority of genome-wide association study discovery has been conducted in populations of European ancestry, and equitable implementation requires targeted multi-ancestry genomic studies [30].

Artificial Intelligence and Machine Learning

AI tools are being evaluated across multiple nodes of the prostate cancer screening pathway. In imaging, deep-learning algorithms trained on mpMRI datasets have demonstrated performance comparable to expert radiologists in internal validation studies; however, external generalizability across scanner platforms and reader populations requires prospective confirmation [13,14]. In pathology, AI systems applied to digitized whole-slide images have shown concordance with expert uropathologists in Gleason grading, with some systems demonstrating reduced inter-observer variability compared with human assessment [14]. Multi-modal AI models integrating PSA, imaging features, clinical variables, and molecular biomarkers are under development, but prospective clinical validation and regulatory approval are prerequisites before widespread deployment [13,14].

Liquid Biopsy and Emerging Molecular Tools

Circulating tumor DNA (ctDNA)-based assays are being explored as non-invasive complements to tissue-based diagnostics, primarily in the setting of advanced and metastatic prostate cancer; their role in early detection of localized disease remains investigational [13]. Urine exosome-based tools, such as the ExoDx Prostate IntelliScore, have been evaluated in prospective studies for their ability to stratify biopsy need in men with PSA 2-10 ng/mL presenting for initial biopsy [24]. While such tools expand the repertoire of non-invasive risk assessment, their integration into routine clinical pathways requires further validation and health economic evaluation.

Clinical Implications and Recommendations

PSA testing should be preceded by an SDM conversation that explains potential benefits and harms in language accessible to the individual patient, with documentation of the discussion [10-12]. The content and quality of this counselling, rather than simply the ordering of a test, constitutes genuine SDM.

A single elevated PSA result should not automatically trigger biopsy. The AUA/SUO recommends confirming a newly elevated PSA with a repeat measurement before proceeding to secondary biomarker testing, imaging, or biopsy [12]. Following confirmation, pre-biopsy mpMRI and selective use of validated biomarkers such as PHI or 4Kscore can refine biopsy decisions; sequential approaches incorporating these tools have been associated with reduced rates of unnecessary biopsy [19-22].

Risk stratification should be tailored to individual patient characteristics. The AUA/SUO and EAU support earlier screening discussion for men at increased risk, including those of African ancestry, those with a first-degree relative diagnosed with prostate cancer at a young age, and known carriers of high-risk germline variants [11,12]. For men at average risk, AUA/SUO recommends baseline PSA discussion beginning at age 45, while the USPSTF focuses individualized screening on men aged 55 to 69 years [10,12].

AS should be discussed as the preferred management option for most men with very-low-risk or low-risk prostate cancer and for appropriately selected men with favorable intermediate-risk disease [11,12]. The 15-year ProtecT outcomes confirm that prostate cancer-specific mortality remained low across treatment strategies for men with PSA-detected localized disease, reinforcing the safety of deferring treatment in eligible patients [27].

Clinicians should note that the implementation of novel biomarker and genomic tools should follow regulatory validation and be accessible across diverse patient populations. Current evidence indicates that PRS-based tools have been primarily validated in European-ancestry cohorts, and extrapolation to other populations requires caution pending further multi-ancestry validation [30].

Clinical risk stratification calculators represent an important practical tool for integrating multiple variables into individualized biopsy risk estimates. The ERSPC Risk Calculator (available at prostatecancer-riskcalculator.com) incorporates PSA, DRE result, prostate volume, transrectal ultrasound findings, and prior biopsy status to generate a percentage risk of prostate cancer on biopsy, and it has been validated across multiple European cohorts. The Prostate Cancer Prevention Trial (PCPT) Risk Calculator similarly combines clinical variables with PSA to estimate biopsy risk. These calculators are recommended by the EAU as decision-support tools prior to biopsy, enabling a more nuanced risk assessment than PSA threshold alone and potentially reducing unnecessary biopsies in men at low calculated risk [11]. Their routine incorporation into clinical pathways represents a practical, low-cost step toward individualized screening that does not require advanced biomarker testing.

Limitations

This review has several limitations. As a narrative rather than a systematic review, the selection of evidence reflects editorial judgement and may not capture all relevant studies. No formal quantitative synthesis or meta-analysis was performed; findings are summarized narratively given the heterogeneous nature of the evidence base. The literature search prioritized English-language PubMed-indexed publications and may not fully represent research published in other languages or databases. Given the rapid evolution of biomarker and AI evidence, some findings described here may be superseded by studies published after the search date of March 2025. The evidence base for several emerging approaches including PRSs in non-European populations and AI-assisted diagnostics in prospective clinical settings remains limited. No formal risk-of-bias assessment was performed for individual included studies.

## Conclusions

PSA-based screening in organized population programs shifts diagnosis toward earlier-stage disease and may reduce prostate cancer-specific mortality, although the magnitude of benefit remains modest and applies specifically to population-level cohorts; for individual patients presenting opportunistically to a physician, the benefit-harm calculation differs and must be contextualized within an SDM framework that accounts for personal risk, values, and clinical circumstances. It simultaneously generates overdiagnosis and overtreatment that impose well-characterized quality-of-life harms, and differences among international guidelines reflect varying interpretations of this benefit-harm balance. Current evidence increasingly supports risk-adapted and individualized approaches embedding PSA within a broader framework incorporating patient age, ancestry, family history, germline genetic risk, validated biomarkers, and pre-biopsy imaging rather than universal threshold-based screening.

Emerging approaches including genomic risk stratification, AI-assisted diagnostics, and liquid-biopsy technologies remain under active investigation and may contribute to future individualized screening strategies if prospective clinical validation is achieved. Delivering these advances equitably, across populations of diverse ancestry and varying healthcare access, remains a principal challenge and a clinical imperative in the early detection of prostate cancer.
